# Novel Autologous Regulatory T-Cell Therapy Ameliorates DSS-Induced Colitis in Humanized Mice

**DOI:** 10.1093/ibd/izaf141

**Published:** 2025-07-07

**Authors:** Md Jabed Khan, Yoo Jin Lee, Su Yeon Lee, Hyeyeon Chung, Thuy Nguyen-Phuong, Yong-Hee Kim, Chung-Gyu Park, Young Mo Kang

**Affiliations:** Preclina Inc., 719 & 1302, Teratower B, 167, Songpa-daero, Songpa-gu, Seoul, South Korea; Division of Rheumatology, Department of Internal Medicine, Kyungpook National University School of Medicine, Daegu, South Korea; Preclina Inc., 719 & 1302, Teratower B, 167, Songpa-daero, Songpa-gu, Seoul, South Korea; Preclina Inc., 719 & 1302, Teratower B, 167, Songpa-daero, Songpa-gu, Seoul, South Korea; Department of Biomedical Sciences, College of Medicine, Seoul National University, Seoul, South Korea; Transplantation Research Institute, Medical Research Center, Seoul National University, Seoul, South Korea; Department of Biomedical Sciences, College of Medicine, Seoul National University, Seoul, South Korea; Transplantation Research Institute, Medical Research Center, Seoul National University, Seoul, South Korea; Department of Microbiology and Immunology, College of Medicine, Seoul National University, Seoul, South Korea; Cancer Research Institute, Seoul National University, Seoul, South Korea; Department of Basic Research, PB Immune Therapeutics Inc, Seoul, South Korea; Department of Biomedical Sciences, College of Medicine, Seoul National University, Seoul, South Korea; Transplantation Research Institute, Medical Research Center, Seoul National University, Seoul, South Korea; Department of Microbiology and Immunology, College of Medicine, Seoul National University, Seoul, South Korea; Cancer Research Institute, Seoul National University, Seoul, South Korea; Department of Basic Research, PB Immune Therapeutics Inc, Seoul, South Korea; Preclina Inc., 719 & 1302, Teratower B, 167, Songpa-daero, Songpa-gu, Seoul, South Korea; Division of Rheumatology, Department of Internal Medicine, Kyungpook National University School of Medicine, Daegu, South Korea

**Keywords:** inflammatory bowel disease, humanized mice, dextran sodium sulfate colitis, regulatory T cells, autologous treg therapy, preclinical model

## Abstract

**Background:**

Inflammatory bowel disease (IBD) is a chronic inflammatory disorder with a complex immune-mediated pathogenesis. The efficacy of human-specific cellular immunotherapies and biological medications cannot be accurately evaluated using traditional murine IBD models. Therefore, a humanized mouse model of IBD is necessary. Regulatory T cells (Tregs) are critical for maintaining intestinal immune homeostasis and may have therapeutic potential for treating IBD.

**Methods:**

Donor peripheral blood mononuclear cells (PBMCs) were used to reconstitute the human immune system in NOG mice and for Treg isolation. T cells were sorted and stimulated with anti-CD3 and anti-CD28 in the presence of irradiated feeder cells to prepare Treg cells. Two weeks after PBMC reconstitution in NOG mice, colitis was induced with dextran sodium sulfate (DSS). The expanded Treg cells were administered intravenously. Ozanimod was used as a positive control.

**Results:**

After expansion, 65.4% of the live CD4^+^ cells were Foxp3^+^CD25^+^ Treg cells and 14.5% were non-Treg cells. The mean human leukocyte (hCD45^+^) engraftment rate in the humanized mice was 56.5% ± 4.5%. Autologous Treg-cell therapy significantly reduced the disease activity index by 78% on day 7. Colonic length was preserved, and colonic inflammation was reduced in mice treated with Treg cells. Immunohistology revealed reduced human T-cell infiltration in Treg-treated mice.

**Conclusions:**

Autologous Treg therapy ameliorated the symptoms of DSS-induced colitis in a humanized mouse model. The autologous PBMC-humanized DSS-induced colitis model may serve as a robust preclinical platform for evaluating the efficacy of personalized Treg cell therapy for IBD.

## Graphical Abstract

**Graphical Abstract:**
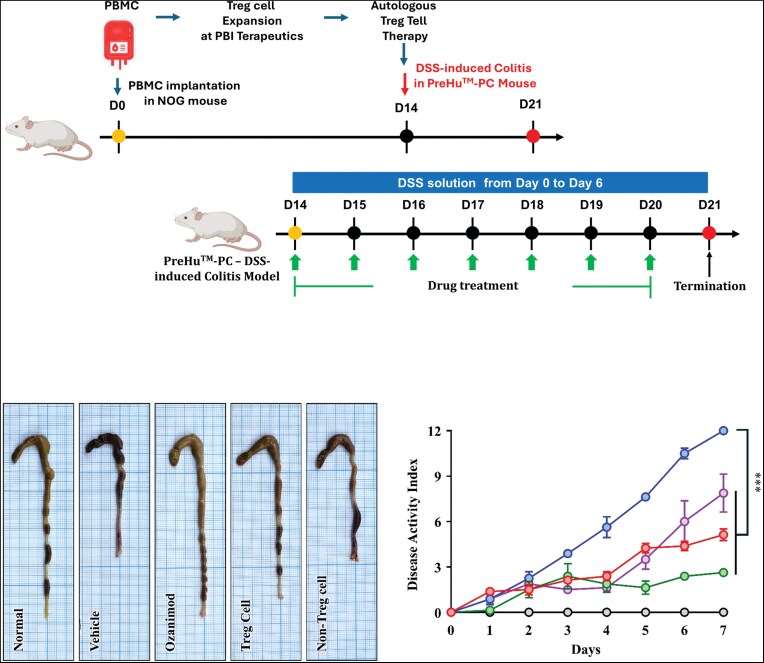

**Key Messages**

- Regulatory T cells (Tregs) play a crucial role in immune homeostasis and have therapeutic potential in inflammatory bowel disease (IBD). However, conventional murine models cannot accurately assess human-specific immunotherapies.
- This study establishes a peripheral blood mononuclear cell (PBMC)-humanized mouse model of dextran sodium sulfate–induced colitis and demonstrates that autologous Treg therapy significantly reduces disease severity by modulating human immune responses.
- By providing a clinically relevant preclinical model, this study supports the development of personalized Treg-based therapies for IBD, offering a potentially safer and more effective treatment strategy for patients.

## Introduction

Inflammatory bowel disease (IBD), which includes ulcerative colitis (UC) and Crohn’s disease (CD), is a chronic inflammatory disorder of the gastrointestinal tract affecting millions of individuals worldwide.^1^ UC is characterized by mucosal inflammation of the colon, and CD is characterized by transmural inflammation occurring anywhere in the gastrointestinal tract.^2^ Interactions between genetic predisposition, environmental triggers, and dysregulated immune responses cause IBD.^3,4^ Disruption of the epithelial barrier, exposing the underlying tissue to luminal antigens, triggers an aberrant T-cell-mediated immune response against commensal bacteria, leading to chronic inflammation and intestinal damage.^5^

Regulatory T cells (Tregs), a key regulator of immune homeostasis, suppress excessive immune responses to control intestinal inflammation. Tregs modulate proinflammatory pathways, help maintain intestinal immune tolerance, and promote mucosal healing.^6^ Studies in FoxP3-deficient scurfy mice and patients with immune dysregulation, polyendocrinopathy, enteropathy, X-linked syndrome (IPEX) syndrome demonstrate the significant role of Tregs in IBD; the absence of functional Tregs due to dysregulated T-cell activation leads to severe intestinal inflammation.^7,8^ Treg depletion exacerbates intestinal inflammation in experimental colitis models, and the adoptive transfer of Tregs alleviates disease severity and promotes tissue repair.^9^ Autologous Treg therapy, involving isolating, expanding, and administering the expanded Tregs from the same individual, is a novel approach to restoring immune balance. This strategy, which minimizes the risks of immune rejection and enhances therapeutic compatibility, is well suited for immune-mediated diseases like IBD. Preclinical studies demonstrated that the adoptive transfer of in-vitro-expanded Tregs reduces inflammation and improves histological outcomes in murine acute graft-versus-host disease (GvHD) and airway allergic inflammation models.^10,11^

Experimental models that closely mimic human IBD are essential for investigating the efficacy of autologous Treg therapy. Conventional murine models, such as the dextran sodium sulfate (DSS)-induced colitis model, have been instrumental in elucidating IBD pathogenesis and assessing potential therapies.^12^ However, mouse immune systems do not adequately replicate human-specific immune responses. Thus, biologics such as monoclonal antibodies and cellular immunotherapies cannot be evaluated using conventional models.^13,14^ To address the limitations of traditional IBD models, humanized mouse models, involving the engraftment of human hematopoietic stem cells (HSCs) or peripheral blood mononuclear cells (PBMCs) into immunodeficient mice, have been developed.^15,16^ Advances in genetically engineered mice, such as the NOG, NSG, and BRG mouse strains, have further enhanced the utility of humanized models in translational research.^17^

Humanized mouse models are invaluable in studying immune-mediated diseases and evaluating therapeutic interventions such as Treg therapy.^15,18^ Humanized mice engrafted with HSCs or PBMCs have been used to study colitis induced by oxazolone or trinitrobenzene sulfonic acid (TNBS) and allergen-induced intestinal inflammation.^19–24^ However, DSS-induced colitis, a widely used reproducible model resembling human UC,^25^ has not been extensively studied in humanized mice. This study aimed to address this gap by establishing a DSS-induced colitis model in humanized NOG mice and evaluating the therapeutic potential of autologous Treg therapy. The novelty of this approach lies in isolating and expanding Tregs from the same donors used to reconstitute the mice to ensure immune compatibility and enhance translational relevance. We hypothesized that autologous Treg therapy will mitigate inflammation, restore intestinal barrier integrity, and improve disease outcomes by modulating human-specific immune responses. Ozanimod, a selective sphingosine-1-phosphate receptor modulator with anti-inflammatory effects in UC, was used as a positive control.^26^ The results of this study improve our understanding of Treg-mediated immune regulation and establish a robust platform for evaluating human-specific therapies in IBD.

## Materials and Methods

### Animals and Ethical Compliance

All animal care and experimental procedures were approved by the Kyungpook National University Institutional Animal Care and Use Committee and conducted according to the institutional guidelines and the ARRIVE guidelines for reporting animal research.^27,28^ Adult CIEA NOG (NOD.Cg-Prkdcscid IL2γtm1Sug/JicKoat) mice were purchased from Koatech. The mice were housed in ventilated micro-isolator cages (maximum of 3 per cage) with corn cob bedding, which was changed twice a week. The controlled environment included a 12-hout light–dark cycle, a temperature of 20°C ± 2°C, and a relative humidity of 50% ± 20%. Mice had ad libitum access to commercial food pellets and water. Mice were euthanized at the end of the study using controlled CO_2_ inhalation in compliance with institutional euthanasia protocols.

### Isolation of PBMCs

After obtaining written informed consent and Ethics Committee approval from Kyungpook National University Hospital, peripheral blood samples were collected from healthy donors in ethylenediaminetetraacetic acid (EDTA)-coated tubes to isolate PBMCs. For each experimental cohort, PBMCs from a single donor were used both for reconstituting the humanized mice and for expanding autologous Treg cells for therapeutic administration. This ensured that all treatments were strictly autologous, with no cross-donor transfer, thereby eliminating inter-donor variability within treatment groups. Blood was diluted with Dulbecco’s phosphate-buffered saline (PBS) and layered over Ficoll–Paque at a 3:5 ratio. After centrifugation at 500 *g* for 30 minutes, the PBMC layer was carefully aspirated and washed twice with RPMI 1640 medium. Cell viability and counts were determined using Trypan Blue exclusion and an Invitrogen Countess 3 automated cell counter, respectively. PBMCs were cryopreserved in 20% freezing media at a concentration of 3.3 × 10^7^ cells per vial and stored in liquid nitrogen.

### Generation of Humanized Mice

Adult female NOG mice (6 weeks) were used for PBMC humanization. Cryopreserved PBMCs from healthy donor blood samples were thawed and 1 × 10^7^ PBMCs were resuspended in 100 µL of PBS for each mouse. Mice were gently restrained, and the resuspended PBMCs were injected into the tail vein using a 100-µL insulin syringe. Mice were briefly observed after the injection to ensure successful PBMC administration and then returned to their cages for routine care and monitoring. Blood samples were collected on day 14 via retro-orbital bleeding using sodium-heparinized capillary tubes for engraftment rate analysis. Blood samples were collected via cardiac puncture at the end of the study. Blood was processed using RBC lysis buffer for downstream analysis.

### DSS-Induced Colitis Model

After confirming the successful humanization of the mice using blood FACS analysis, acute colitis was induced in humanized mice using DSS (36–40 kDa; MP Biomedicals). The freshly prepared DSS solution was administered to mice for 7 consecutive days, replacing the solution every other day. The DSS regimen used in this study was selected based on established protocols and internal pre-validation to ensure the reproducibility of acute colitis induction in humanized NOG mice while minimizing systemic toxicity. Throughout the treatment period, Clinical assessments, including body weight, stool consistency, and intestinal bleeding, were performed daily, and the disease activity index (DAI) was calculated by summing these scores (Table S1). Mice were euthanized on day 7, and the colons were excised for further analysis, including length and weight (with and without stool) measurements and histological and molecular tissue analyses. The experimental groups included: Normal (PBMC-humanized mice without DSS-induced colitis), Vehicle (PBMC-humanized mice with DSS-induced colitis, and treated with PBS), Ozanimod (PBMC-humanized mice with DSS-induced colitis and treated with 5 mg/kg Ozanimod), Autologous Treg (PBMC-humanized mice with DSS-induced colitis and treated with 5 × 10^6^ expanded autologous Treg cells), non-Treg (PBMC-humanized mice with DSS-induced colitis and treated with 5 × 10^6^ autologous CD4^+^CD25^−^ effector T cells), and NOG-Vehicle (non-humanized NOG mice with DSS-induced colitis, without PBMC engraftment). The group size (*N* = 4) was determined based on technical feasibility, as PBMCs from each donor were used for both humanizing the mice and expanding autologous Tregs. This design required large quantities of donor cells per cohort and limited the number of animals per group. No animals were lost due to DSS-related mortality, and all mice meeting humanization criteria were included in the final analysis.

### Autologous Treg Cell Expansion and Treatment

Tregs were isolated from donor PBMCs using the EasySepTM Human CD4^+^ and CD25^+^ regulatory T-cell isolation kits (STEMCELL Technologies). The isolated Tregs were expanded ex vivo in X-VIVO 15 medium supplemented with 500 IU/mL IL-2 and activated with anti-CD3/CD28-coated beads at a 1:1 bead-to-cell ratio. Irradiated autologous PBMCs (30 Gy) were used as feeder cells to support Treg proliferation. Expansion was conducted over two 7-day rounds, and cells were frozen between rounds. The phenotype and quality of the expanded Tregs were assessed by flow cytometry using the following markers: CD3, CD4, CD25, CD127, FoxP3, PD-1, CTLA-4, Helios, and RORγt. Non-Treg cells (CD4^+^CD25^−^) isolated during the sorting process were cultured and analyzed in parallel as controls. On day 0 of colitis induction, the expanded Tregs were resuspended in sterile PBS and 5 × 10^6^ cells/100 µL were administered intravenously via tail vein injection. The control groups received non-Treg cells or Vehicle (PBS) alone.

### Flow Cytometry

Single-cell suspensions from peripheral blood, spleen, and bone marrow were stained with antibodies (Table S2). Cells were blocked with human Fc receptor blocking buffer and incubated with primary antibodies for 30 minutes at 4°C. After washing, the cells were fixed in 2% paraformaldehyde and analyzed using a NovoCyte Flow Cytometer (ACEA Biosciences). Immune cell populations were quantified using Novoexpress software.

### Histological Analysis

Colons were arranged as Swiss rolls and fixed in 10% neutral-buffered formalin for 24 hours. Samples were processed using a Tissue-Tek VIP 6 Tissue Processor (Sakura), embedded in paraffin, and sectioned at 3-µm thickness using a microtome (Leica Biosystems). Colon sections were deparaffinized with xylene, rehydrated with graded ethanol, and stained with hematoxylin and eosin (H&E) following standard protocols. Stained slides were scanned using a digital slide scanner, and images were captured using the slide scanner software. Four representative fields per sample were scored by 2 blinded independent researchers. Histological scoring was performed as previously described^29^ to assess inflammation, crypt damage, and the extent of tissue involvement (Table S3).

### Immunofluorescence and Immunohistochemical Staining

Immunofluorescence and immunohistochemical staining were performed on formalin-fixed, paraffin-embedded (FFPE) colon sections to visualize Tregs and immune cell infiltration. The expression of Tregs (hCD3^+^FoxP3^+^) was assessed using the Opal Multiplex Immunohistochemistry Assay Kit (Akoya Biosciences). Sections were dewaxed, rehydrated, and subjected to heat-induced antigen retrieval with Tris-EDTA buffer (pH 9.0) for 10 minutes. After blocking with 5% normal swine serum, the sections were incubated with FoxP3 and hCD3 antibodies for 1 hour followed by incubation with HRP-conjugated secondary antibody for 30 minutes. Signal amplification was achieved using Opal 690 and Opal 520 TSA. The slides were counterstained with DAPI, mounted, and imaged at 400× magnification using a Nikon confocal microscope. Positive cells were manually counted in 4 high-power fields (HPFs) using ImageJ.

For immunohistochemical staining, FFPE sections were dewaxed, rehydrated, and subjected to antigen retrieval using citrate buffer (pH 6.0) for 10 minutes. Sections were blocked with 5% normal swine serum and incubated with hCD45, hCD3, and MPO antibodies for 1 hour at room temperature. After incubation with biotin-labeled secondary antibodies for 30 minutes, the signal was amplified with the ABC kit (Vector Laboratories). Diaminobenzidine was applied as the chromogen, and the sections were counterstained with hematoxylin, dehydrated, cleared in xylene, and mounted. Images were captured at 400× magnification using the Motic DSAssistant software. Positive cells were counted in 4 HPFs per sample using ImageJ, and the results were expressed as the number of positive cells per HPF. A complete list of primary antibodies used is provided in Table S4.

### Statistical Analyses

Data are expressed as means ± SEMs. Two groups were compared using 2-tailed Student’s *t*-test and multiple groups were compared using 1-way analysis of variance (ANOVA) with Tukey’s post hoc test. Correlations were evaluated using Pearson’s correlation coefficient. Statistical significance was set at *P* < .05. GraphPad Prism software was used for graph generation, and IBM SPSS Statistics (v26.0) was used for data analyses.

## Results

### Development of the Humanized Mice

**Figure 1.**
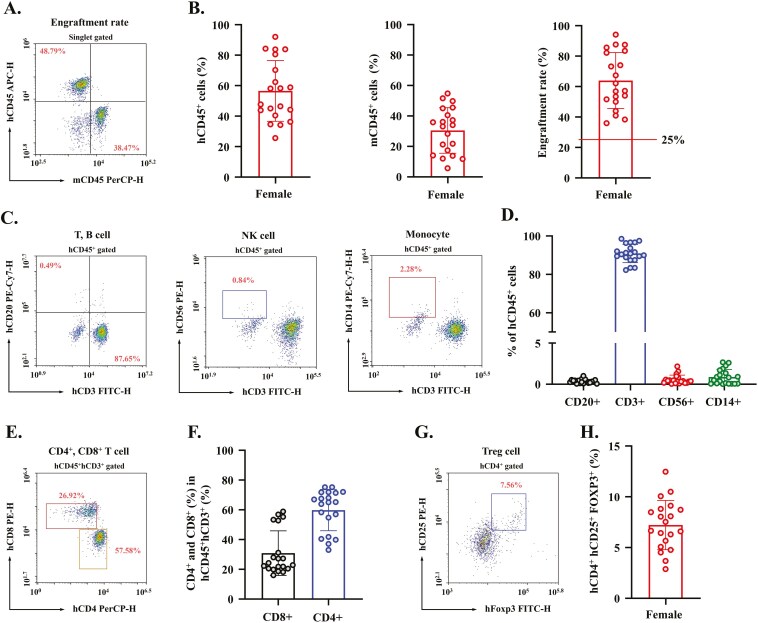
Validation of humanization using FACS analysis of peripheral blood in PBMC-humanized mice. (A) Representative flow cytometry gating plot illustrating the engraftment rate 2 weeks after transplantation of peripheral blood mononuclear cells (PBMCs). (B) Quantification of human and mouse CD45^+^ cells and the engraftment efficiency, expressed as the percentage of human CD45^+^ cells within the total CD45^+^ (human and mouse) population in the peripheral blood 2 weeks after transplantation. (C) Representative flow cytometry gating plots demonstrating human immune system multilineage development 2 weeks after transplantation. The human leukocyte subsets analyzed included T cells (CD3^+^), B cells (CD20^+^), natural killer (NK) cells (CD56^+^), and monocytes (CD14^+^) within the hCD45^+^ population. (D) Quantification of the immune cell subsets within the hCD45^+^ population. (E) Representative flow cytometry gating plot showing the T-cell subsets (CD4^+^ and CD8^+^) within the hCD45^+^CD3^+^ population. (F) Quantification of T-cell subsets as a percentage of the hCD45^+^CD3^+^ population. (G) Representative flow cytometry gating plot of regulatory T cells (Tregs; hCD4^+^hCD25^+^FOXP3^+^) as a proportion of the total hCD3^+^hCD4^+^hCD25^+^ population. (H) Quantification of Treg+ cells within the hCD4^+^hCD25^+^ population. All data are presented as mean ± SEM (*N* = 20). These 20 samples represent humanized mice pooled across all experimental groups in the study.

Thirteen days after the injection of PBMCs from healthy donors into immunodeficient NOG mice, peripheral blood samples were analyzed via flow cytometry to confirm the engraftment of human immune cells and the reconstitution of human immune system components. The flow cytometry analysis revealed that 56.53% of singlet-gated cells expressed hCD45 (Figure 1A and 1B), signifying a robust presence of human leukocytes in the peripheral blood of the humanized mice. T cells (hCD3^+^) accounted for 90.88% of the hCD45^+^ population, and 0.36% of hCD45^+^ cells were B cells (hCD20^+^) (Figure 1C and 1D), indicating a predominance of T-cell-mediated immunity in the engrafted mice. In addition to the T and B cells, 0.56% of the hCD45^+^ cells were natural killer (NK) cells, confirming the partial reconstitution of innate immune components, and 0.88% of the hCD45^+^ population were monocytes, which are essential for phagocytic and antigen-presenting functions (Figure 1C and 1D). CD4^+^ helper T cells (59.82%) and CD8^+^ cytotoxic T cells (30.90%) were successfully engrafted, highlighting the reconstitution of both helper and cytotoxic adaptive immune compartments (Figure 1E and 1F). Additionally, 7.22% of the hCD45^+^ T cells were regulatory T cells (hCD4^+^hCD25^+^ hFoxP3^+^) (Figure 1G, 1H), indicating the potential for functional immune modulation in the humanized mice. Overall, these results demonstrate the successful engraftment of human PBMCs into NOG mice, resulting in a robust reconstitution of the human immune system components, including T cells, B cells, NK cells, monocytes, and Tregs. This humanized mouse model provides a platform for the induction of DSS-induced colitis to study the therapeutic efficacy of autologous Tregs.

To further assess the phenotypic quality of the expanded Tregs, flow cytometry analysis was performed on the expanded cells. The results confirmed that 65.4% of live CD4^+^ cells were FoxP3^+^CD25^+^ Treg cells, while 14.5% were CD4^+^CD25^−^ non-Tregs (Figure S1C and S1D). To ensure accurate gating and minimize false-positive signals, a FoxP3 fluorescence-minus-one control was included, establishing the threshold for FoxP3^+^ expression (Figure S1B). The high proportion of FoxP3^+^CD25^+^ cells indicates a highly enriched population of functionally suppressive Tregs, supporting their suitability for therapeutic application in DSS-induced colitis.

### Autologous Treg-Cell Therapy Ameliorated the Symptoms of DSS-Induced Colitis

**Figure 2.**
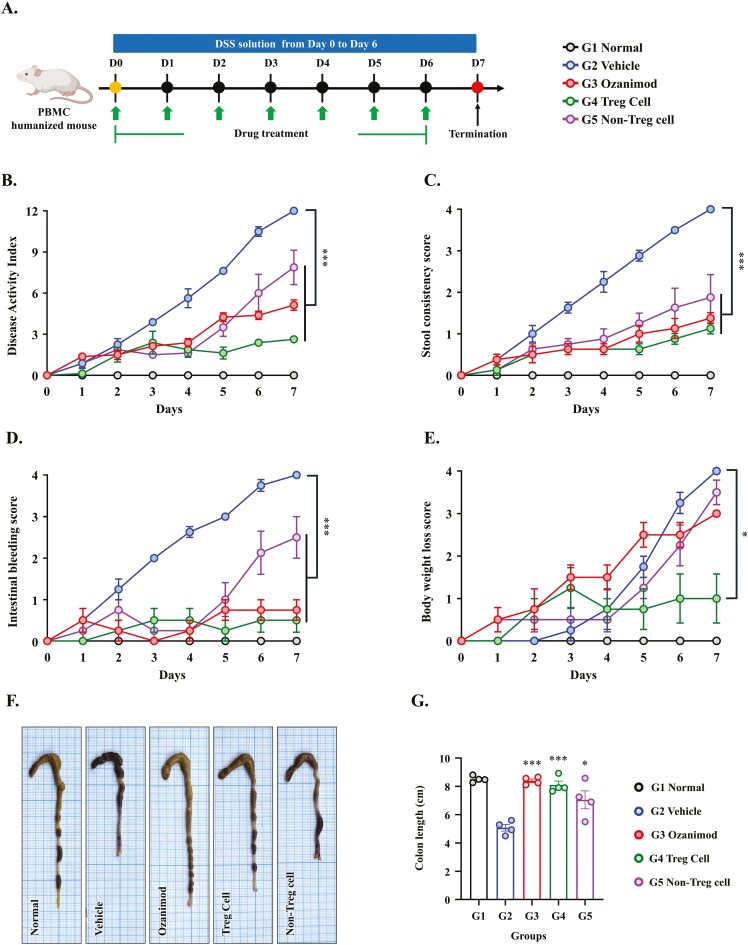
Autologous regulatory (Treg)-cell therapy ameliorates the clinical symptoms of DSS-induced colitis in PBMC-humanized mice. (A) Schematic representation of the experimental timeline for dextran sodium sulfate (DSS)-induced colitis. Mice received DSS solution in drinking water from day 0 to day 6. Treatments were initiated during DSS exposure as indicated: Normal (PBMC-humanized mice without DSS-induced colitis), Vehicle (PBMC-humanized mice with DSS-induced colitis, and treated with phosphate-buffered saline [PBS]), Ozanimod (PBMC-humanized mice with DSS-induced colitis and treated with 5 mg/kg Ozanimod), Autologous Treg (PBMC-humanized mice with DSS-induced colitis and treated with 5 × 10^6^ expanded autologous Treg cells), Non-Treg (PBMC-humanized mice with DSS-induced colitis and treated with 5 × 10^6^ autologous CD4^+^CD25^−^ effector T cells). (B) disease activity index (DAI), (C) stool consistency score, (D) intestinal bleeding score, and (E) body weight loss scores for the different treatment groups. DAI was calculated as the sum of stool consistency, intestinal bleeding, and body weight loss scores. (F) Representative images of colons from each treatment group demonstrating macroscopic changes. (G) Colon length as a measure of colitis severity. Data are presented as mean ± SEM (*N* = 4 per group). *P < .05, **P < .01, ***P < .001 versus the Vehicle group.

After successful humanization, colitis was induced by administering DSS in the drinking water for 6 days (Figure 2A). The following experimental groups were included in the final therapeutic comparison: Normal (PBMC-humanized mice without DSS-induced colitis), Vehicle (PBMC-humanized mice with DSS-induced colitis, and treated with PBS), Ozanimod (PBMC-humanized mice with DSS-induced colitis and treated with 5 mg/kg Ozanimod), Autologous Treg (PBMC-humanized mice with DSS-induced colitis and treated with 5 × 10^6^ expanded autologous Treg cells), and Non-Treg (PBMC-humanized mice with DSS-induced colitis and treated with 5 × 10^6^ autologous CD4^+^CD25^−^ effector T cells). The therapeutic effects of autologous Treg-cell therapy, Ozanimod (positive control), Non-Treg cell treatment, and Vehicle were compared. The DAI scores (Figure 2B) were highest in the Vehicle-treated group, reflecting severe clinical symptoms. Treg therapy significantly reduced DAI scores compared with the Vehicle-treated group (2.6 ± 0.3 vs. 12.0 ± 0.0, *P* < .001), outperforming both ozanimod (5.1 ± 0.4, *P* < .001) and Non-Treg cells (7.8 ± 1.2, *P* < .001). Stool consistency and intestinal bleeding scores (Figure 2C and 2D) followed a similar trend, showing improved outcomes in the Treg-treated group. Body weight changes (Figure 2E) indicated that Vehicle-treated mice experienced significant weight loss compared with normal mice. Treg therapy effectively mitigated weight loss, with body weights in the Treg treatment group remaining close to those of normal mice. Treatment with ozanimod and Non-Treg cells resulted in considerable weight loss. Mean colon length was significantly shorter in the Vehicle-treated group compared with the Treg and ozanimod treatment groups (Figure 2G) (5.1 ± 0.2 cm vs. 8.1 ± 0.2 cm and 8.3 ± 0.1 cm, respectively; *P* < .001 for both). Non-Treg cell treatment partially attenuated colon shortening (7.1 ± 0.6 cm, *P* < .05). Representative colon images (Figure 2F) demonstrate that structural integrity was preserved in the Treg treatment group, whereas the Vehicle group exhibited severe damage and shortening. To assess whether differences in disease severity could be attributed to variability in immune reconstitution, we compared mean hCD45^+^ engraftment rates across the experimental groups. The Treg group exhibited a mean engraftment rate of 57.5%, comparable to that of the Non-Treg (67.2%), Ozanimod (63.5%), and Vehicle (54.3%) groups (Table S5). Despite these similar engraftment levels, the Treg-treated group displayed markedly reduced DAI scores compared to all other DSS-treated groups, indicating that the therapeutic benefit was not driven by differences in hCD45^+^ engraftment efficiency. In summary, autologous Treg-cell therapy mitigated DSS-induced colitis in PBMC-humanized mice. Clinical parameters, including body weight, DAI scores, and colon morphology, were significantly improved in response to Treg therapy, underscoring its therapeutic potential for treating colitis.

In a preliminary pilot experiment, we also evaluated DSS-induced colitis in non-humanized NOG mice (NOG-Vehicle group), which exhibited similar disease severity compared to humanized mice (Figures S2 and S3). These data further support the robustness of our colitis induction protocol.

### Autologous Treg-Cell Therapy Reduced Inflammation in DSS-Induced Colitis

**Figure 3.**
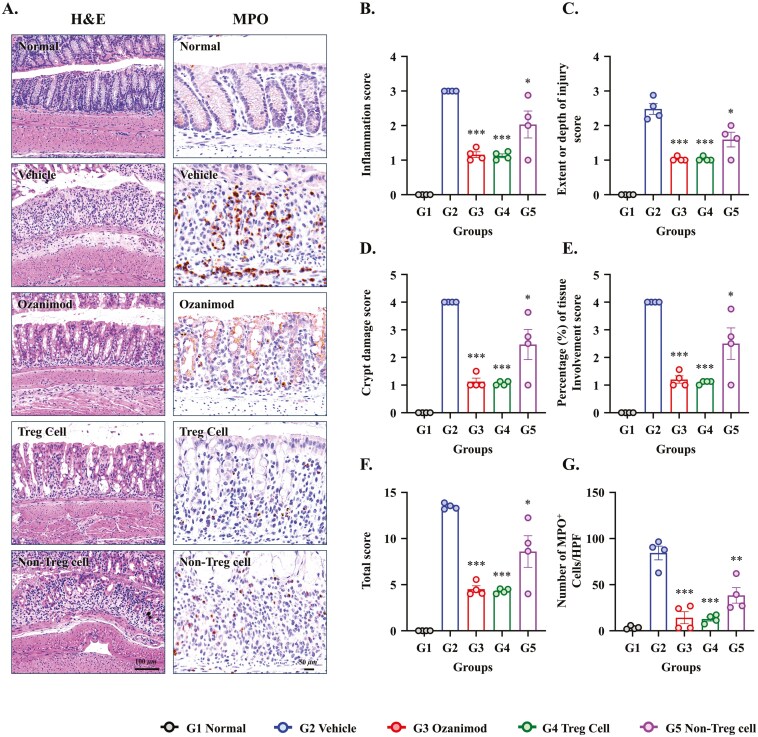
Histopathological and immunohistochemical evaluation of colon tissue in the dextran sodium sulfate (DSS)-induced colitis model. (A) Representative images of hematoxylin and eosin (H&E) and MPO staining of colon tissue sections from peripheral blood mononuclear cell (PBMC) humanized mice after 7 days of treatment. The treatment groups included Normal, Vehicle, Ozanimod (5 mg/kg), autologous regulatory T (Treg) cells, and Non-Treg cells. Scale bars: 100 μm (H&E) and 50 μm (MPO). Semi-quantitative histological scoring for (B) inflammation severity, (C) extent (depth) of injury, (D) crypt damage, (E) percentage of tissue involvement, and (F) total histological score. (G) Quantification of MPO^+^ cells per high-power field (HPF). Data are presented as mean ± SEM (*N* = 4 per group). *P < .05, **P < .01, ***P < .001 versus the Vehicle group.

In PBMC-humanized mice, autologous Treg-cell therapy significantly reduced histological inflammation and crypt damage induced by DSS. Histological evaluation of H&E-stained colon sections revealed stark differences in inflammation and epithelial integrity among the experimental groups (Figure 3A). Vehicle-treated mice exhibited severe inflammation characterized by crypt destruction, epithelial erosion, and substantial infiltration of inflammatory cells. These findings were consistent with the high inflammation scores. Treg cell and ozanimod therapies ameliorated inflammation, reducing inflammation scores compared with the Vehicle group (3.00 ± 0.00 vs. 1.13 ± 0.06 and 1.16 ± 0.08, respectively; *P* < .001 for both, Figure 3B). Non-Treg-cell treatment induced moderate improvements in inflammation scores (2.03 ± 0.39, *P* < .05 vs. Vehicle), indicating partial protection. Crypt damage scores were significantly reduced in the Treg and ozanimod treatment groups compared with the Vehicle-treated group (4.00 ± 0.00 vs. 1.06 ± 0.04 and 1.13 ± 0.13, respectively; *P* < .001 for both, Figure 3D), reflecting attenuation of the extensive epithelial destruction induced by DSS. Non-Treg-cell treatment induced pronounced improvements in crypt damage scores (2.47 ± 0.55, *P* < .05 vs. Vehicle).

To further evaluate the severity of the neutrophil-driven inflammation, MPO-positive cells were quantified as a marker of neutrophil infiltration (Figure 3G). MPO-positive cells were significantly lower in the Treg and ozanimod treatment groups compared with the Vehicle-treated group (84.4 ± 7.4 cells/HPF vs. 12.8 ± 2.2 cells/HPF and 14.2 ± 6.5 cells/HPF, *P* < .001 for both). Non-Treg cells induced partial reductions in MPO-positive cells (38.4 ± 8.5 cells/HPF, *P* < .01 vs. Vehicle). In summary, autologous Treg cell therapy reduced colonic inflammation, preserved crypt architecture, and diminished neutrophil infiltration in mice with DSS-induced colitis.

### Autologous Treg-Cell Therapy Reduces Immune Cell Infiltration in DSS-Induced Colitis

**Figure 4.**
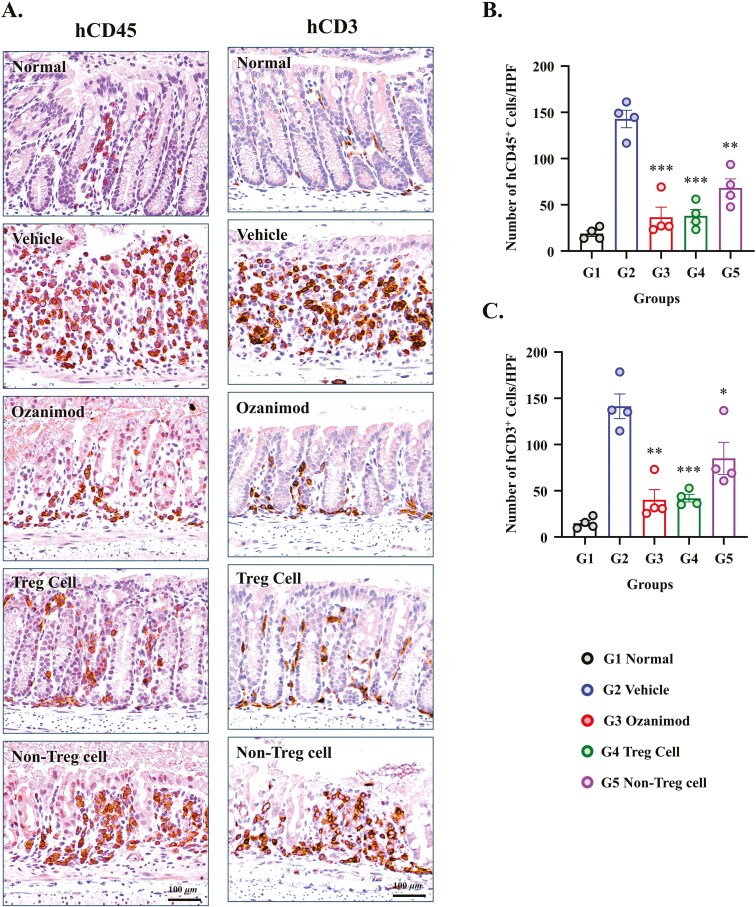
Immunohistochemical analysis of hCD45^+^ and hCD3^+^ cells in the colons of mice with dextran sodium sulfate (DSS)-induced colitis treated with autologous Treg cells. (A) Representative images of hCD45^+^ and hCD3^+^ immunohistochemically stained colon sections from PBMC-humanized mice after 7 days of treatment. The treatment groups included Normal, Vehicle, Ozanimod (5 mg/kg), autologous regulatory T (Treg) cells, and Non-Treg cells. Scale bars: 50 μm. (B) Quantification of hCD45^+^ cells per high-power field (HPF). (C) Quantification of hCD3^+^ cells per HPF. Data are expressed as mean ± SEM (*N* = 4 per group). *P < .05, **P < .01, ***P < .001 versus the Vehicle group.

To evaluate the impact of autologous Treg-cell therapy on immune cell dynamics in mice with DSS-induced colitis, immunohistochemical staining for hCD45 and hCD3 in colonic tissue was performed to assess human immune cell and T-cell infiltration, respectively. Immunohistochemical staining for hCD45 revealed pronounced differences in immune cell infiltration across the groups (Figure 4A). The Vehicle-treated mice exhibited the highest levels of hCD45-positive cells, indicating severe immune-mediated inflammation. In contrast, reduced hCD45-positive cell recruitment was observed in the colons from mice in the Treg treatment group, indicating reduced immune cell infiltration. Ozanimod treatment also attenuated immune cell infiltration, although less effectively than Treg therapy. In the Non-Treg cell treatment group, hCD45-positive cell recruitment was modestly reduced. Normal mice displayed minimal hCD45-positive staining, consistent with the absence of inflammation. Quantitative analysis of hCD45-positive cells (Figure 4B) confirmed these observations. Treg and ozanimod treatments significantly reduced hCD45-positive cells in the colon compared with the Vehicle-treated group (142.9 ± 9.4 cells/HPF vs. 38.0 ± 7.0 cells/HPF and 35.6 ± 10.9 cells/HPF, respectively, *P* < .001 for both). Non-Treg cell therapy resulted in limited decreases in hCD45-positive cells (68.3 ± 9.7 cells/HPF, *P* < .01 vs. Vehicle group).

Immunohistochemical staining for hCD3, a marker of T cells, showed similar trends (Figure 4C). The Vehicle-treated group exhibited the highest levels of hCD3-positive cells, reflecting severe T-cell-driven inflammation. Mice treated with Tregs or ozanimod exhibited significantly reduced hCD3-positive cell infiltration, indicating the suppression of T-cell-mediated immune responses. Non-Treg-cell therapy showed partial effects, and the normal group exhibited minimal hCD3-positive staining, indicating the absence of inflammatory T-cell infiltration. Quantification of hCD3-positive cells (bottom-right graph) corroborated these findings. Treg therapy and ozanimod significantly reduced hCD3-positive cells compared with Vehicle treatment (141.4 ± 13.4 cells/HPF vs. 41.9 ± 4.1 cells/HPF and 40.19 ± 11.0 cells/HPF, respectively; *P* < .001 and *P* < .01, respectively). Non-Treg-cell therapy exhibited partial decreases in hCD3-positive cells (85.0 ± 17.5 cells/HPF, *P* < .05 vs. Vehicle group).

### Immunofluorescence Staining Confirms Increased Treg Infiltration Following Autologous Treg Cell Therapy

**Figure 5.**
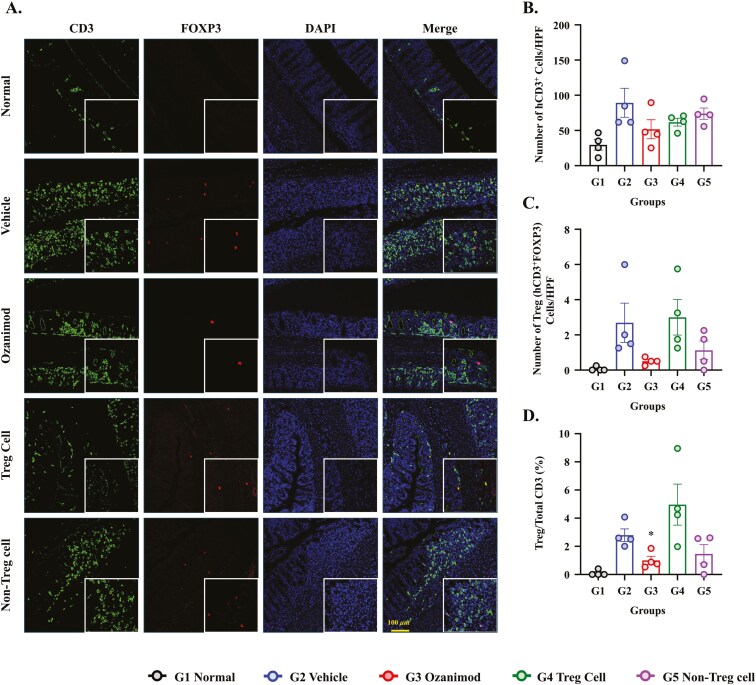
Regulatory T-cell (Treg) immunofluorescence staining of colon sections from the dextran sodium sulfate (DSS)-induced colitis model treated. (A) Representative IF images of Swiss roll colon sections illustrating CD3 and FOXP3 co-expression across treatment groups: normal, Vehicle, ozanimod (5 mg/kg), autologous Treg cells, and Non-Treg cells. DAPI was used to stain nuclei, and merged images show co-localization of CD3^+^FOXP3^+^ Tregs. (B) Quantification of total CD3^+^ cells, (C) CD3^+^FOXP3^+^ Tregs, and (D) the proportion of CD3^+^FOXP3^+^ Tregs within the total CD3^+^ population. Data are presented as mean ± SEM (*N* = 4 per group). *P < .05, **P < .01, ***P < .001 versus the Vehicle group.

Immunofluorescence staining for hCD3^+^FOXP3^+^ Tregs revealed substantial differences in Treg abundance among the experimental groups (Figure 5A). Normal mice exhibited minimal Treg infiltration (0.10% ± 0.10), reflecting the lack of immune activation. Vehicle-treated mice displayed a modest increase in Tregs (2.79% ± 0.45), possibly due to a compensatory response to DSS-induced inflammation. However, these levels were insufficient to counteract the inflammation induced by DSS. Treatment with autologous Tregs markedly enhanced Treg infiltration (4.96% ± 1.45), with increased co-localization of FOXP3^+^ and CD3^+^ T cells. These results highlight the recruitment or retention of functional Tregs at the inflammation site, correlating with therapeutic efficacy. Treatment with ozanimod reduced Treg infiltration (1.00% ± 0.29, *P* < .05 vs. Vehicle), consistent with its immunomodulatory mechanism limiting T-cell migration rather than directly promoting Tregs. Non-Treg-cell therapy resulted in modest Treg infiltration (1.46% ± 0.66), but the levels were significantly lower than the Treg levels in the autologous Treg group. Quantitative analysis of the Treg-positive cell counts supports these findings (Figure 5C). Treg-positive cells/HPF were significantly higher in Vehicle-treated mice (2.69 ± 1.12) and Treg-treated mice (3.00 ± 1.01). Ozanimod-treated mice exhibited fewer Tregs (0.50 ± 0.10), and Non-Treg-cell treatment showed intermediate levels (mean 1.13 ± 0.53). These data confirm the enhancement of Treg recruitment and infiltration in DSS-induced colitis induced by autologous Treg therapy.

## Discussion

This study highlights the efficacy of autologous Treg-cell therapy in mitigating DSS-induced colitis in a humanized NOG mouse model. The humanized immune system was developed to bridge the translational gap between preclinical and clinical research, providing a platform to investigate human-specific immunopathological mechanisms and therapeutic interventions for IBD. Our results demonstrate the therapeutic potential of autologous Tregs in reducing inflammation, restoring intestinal barrier integrity, and alleviating colitis symptoms.

Traditional murine models were pivotal in advancing our understanding of the immunopathology of IBD, the translational applicability of these models was limited due to significant genotypic and phenotypic differences between the murine and human immune systems.^13,30^ To overcome these challenges, PBMCs from healthy donors were engrafted into immunodeficient NOG mice to develop a functional human immune system. Flow cytometric analysis confirmed high levels of human CD45^+^ cell engraftment, exceeding the 50% benchmark of many reported models.^18,31^ Major immune cell subsets, including T cells, B cells, NK cells, monocytes, and regulatory T cells, were detected in the mouse model, indicating a comprehensive multilineage human immune system within the murine host. The predominance of T cells in the reconstituted immune system aligns with the immunopathological mechanisms of IBD; dysregulated T-cell responses are central to disease progression. Notably, Tregs were identified in the hCD45^+^ population, providing a critical baseline for exploring the therapeutic potential of Tregs in modulating colitis-associated inflammation.

Our DSS-induced colitis model, characterized by a robust humanized immune system, a refined DSS protocol, and autologous Treg therapy, is superior to other DSS models in humanized mice, including the models reported in Verhaeghe et al.,^32^ Touch et al.,^33^ and Hofer et al.^34^ This comprehensive model provided a superior platform for studying IBD pathogenesis and evaluating targeted therapeutics, replicating the hallmark clinical features of UC such as weight loss, diarrhea, rectal bleeding, and colon shortening. Histological analysis confirmed the presence of severe epithelial damage, crypt loss, and inflammatory infiltration in the Vehicle-treated mice. These findings highlight the model’s relevance and reliability for assessing therapeutic strategies in an IBD-like context.

Autologous Treg therapy offers unique advantages over other immunosuppressive therapies or biologics, such as anti-TNF agents. Deriving Tregs from the same individual minimizes the risk of immune rejection, ensuring greater compatibility and efficacy. Our results show that autologous Treg therapy significantly reduced clinical disease severity, as evidenced by lower DAI scores and preserved colon morphology compared with Vehicle-treated controls. Unlike broad-spectrum immunosuppressive therapies, which increase the risk of opportunistic infections or severe side effects,^35^ autologous Tregs offer targeted immune modulation, specifically restoring tolerance at the site of inflammation.

Preclinical studies demonstrated that the adoptive transfer of in-vitro-expanded Tregs reduces inflammation and improves histological outcomes in experimental models.^10,11^ Our study extends these findings by demonstrating the efficacy of autologous Tregs in a humanized immune system. Early-phase clinical trials, such as the TRIBUTE study (NCT03185000), support the safety and efficacy of Tregs in Crohn’s disease and demonstrate their potential to improve disease activity and promote mucosal healing.^36^. In our study, the significant infiltration of hCD3^+^FOXP3^+^ Tregs into inflamed colonic tissue strongly correlated with improved clinical outcomes, underscoring the translational relevance of our findings.

Ozanimod, a selective S1P receptor modulator, demonstrated moderate efficacy in alleviating colitis symptoms. Ozanimod reduced the DAI scores and preserved the colon length in our DSS model. However, these effects were less pronounced than the effects of autologous Tregs, especially the effects of DSS on weight loss in the ozanimod group. The limited Treg infiltration observed in ozanimod-treated mice suggests that ozanimod primarily modulates lymphocyte trafficking rather than directly promoting Treg-mediated immunosuppression. These findings highlight the complementary yet distinct mechanisms of ozanimod and Treg therapy.

### Implications and Future Directions

The PBMC-humanized NOG mouse model, while useful for studying human immune responses in vivo, is primarily T-cell–driven and lacks full representation of other human immune compartments, such as B cells and innate immune cells. Similarly, the acute DSS-induced colitis model mainly reflects epithelial injury and innate inflammation, without the chronicity or mucosal remodeling seen in human IBD. These limitations should be considered when translating findings to clinical contexts. Additionally, the relatively small group size (*N* = 4) was constrained by the technical demands of dual PBMC use for humanization and Treg expansion. Despite this, statistically significant treatment effects were observed.

Future studies with chronic colitis models, larger cohorts, and improved humanization strategies—such as stem cell co-engraftment or microbiota introduction—will be essential to validate and extend these findings.

## Conclusions

This study highlights the efficacy of autologous Treg therapy in mitigating DSS-induced colitis in a humanized NOG mouse model. Integrating functional humanized immune systems and a reproducible colitis model provides a robust preclinical platform to evaluate personalized and targeted therapies for IBD. These findings strengthen the growing evidence supporting Treg therapy as a transformative treatment option for IBDs.

## Supplementary Material

izaf141_Supplementary_Material

## Data Availability

The data and materials used in this study are available from the corresponding author upon reasonable request.
