# Supplementary material for: Novel Autologous Regulatory T-Cell Therapy Ameliorates DSS-Induced Colitis in Humanized Mice
Source: Inflamm Bowel Dis. 2025 Jul 7;31(9):2535–46. doi: 10.1093/ibd/izaf141 (PMC12456575; doi:10.1093/ibd/izaf141)
Supplement: izaf141_Supplementary_Material [file izaf141_supplementary_material.docx]

**Novel Autologous Regulatory T Cell Therapy Ameliorates DSS-Induced Colitis in Humanized Mice**

**Author information**

Md. Jabed Khan^1,2,^ PhD, Yoo Jin Lee^2^ PhD, Su Yeon Lee^2^ MS, Hyeyeon Chung^3^ MS, Thuy Nguyen-Phuong^5^ MS, Yong-hee Kim^3,4,5,6,7^ PhD, Chung-Gyu Park^3,4,5,6,7^ PhD, Young Mo Kang^1,2*^ PhD

^1^Division of Rheumatology, Department of Internal Medicine, Kyungpook National University School of Medicine, Daegu, South Korea.

^2^Preclina Inc., 719 & 1302, Teratower B, 167, Songpa-daero, Songpa-gu, Seoul, South Korea.

^3^Department of Biomedical Sciences, College of Medicine, Seoul National University, Seoul, South Korea

^4^Department of Microbiology and Immunology, College of Medicine, Seoul National University, Seoul, South Korea

^5^Transplantation Research Institute, Medical Research Center, Seoul National University, Seoul, South Korea

^6^Cancer Research Institute, Seoul National University, Seoul, South Korea

^7^Department of Basic Research, PB Immune Therapeutics Inc, Seoul, South Korea

*Correspondence:

Young Mo Kang, MD, PhD

CEO

Preclona Inc, Seoul, South Korea

Professor

Division of Rheumatology, Department of Internal Medicine

Kyungpook National University School of Medicine, Daegu, South Korea

Phone No: +82-1566-0536

Fax No: +82-32-218-0536

Email Address: [ymkang@preclina.com](mailto:ymkang@preclina.com)

**Supplementary information**

**Supplementary Table 1: Clinical score of DSS induced colitis model.**

| Score | Body Weight loss score | Stool consistency score | Intestinal bleeding score |
| --- | --- | --- | --- |
| 0 | None | Normal | Negative hemoccult |
| 1 | 1-5% | Soft but still formed | Positive occult blood test  (Weak luminol intensity) |
| 2 | 6-10% | Soft | Positive occult blood test  (Strong luminol intensity) |
| 3 | 11-18% | Very soft; wet | Blood traces in stool visible |
| 4 | > 18% | Watery diarrhea | Gross rectal bleeding |

**Supplementary Table 2: List of antibodies used for FACS analysis.**

| **Antibody** | **Conjugation** | **Company** |
| --- | --- | --- |
| Anti-mouse CD45 | PerCP | Biolegend |
| Anti-human CD45 | APC |  |
| Anti-human CD3 | FITC |  |
| Anti-human CD4 | PerCP |  |
| Anti-human CD8 | PE |  |
| Anti-human CD20 | PE-Cy7 |  |
| Anti-human CD14 | PE-Cy7 |  |
| Anti-human CD56 | PE |  |
| Anti-human FoxP3 | FITC |  |
| Anti-human CD25 | PE |  |

**Supplementary Table 3: Histological scoring for DSS-induced colitis model.**

| Score | Inflammation | Extent or depth of injury | Crypt damage | Percentage (%) of tissue involvement |
| --- | --- | --- | --- | --- |
| 0 | None | None | No Damage | None |
| 1 | Slight (Small, focal, or widely separated, limited to lamina propria) | Mucosal | Basal 1/3 Damage (Loss of bottom one-third of the crypts) | 1-25% (Up to 25% of the tissue effected by disease process) |
| 2 | Moderate (Multifocal or locally extensive, extending to submucosa) | Mucosal and submucosal (Mural) | Basal 2/3 Damage  (Loss of bottom two-third of the crypts) | 26-50%  (Up to 50% of the tissue effected by disease process) |
| 3 | Severe (Transmural inflammation with ulcers covering >20 crypts) | Transmural  (Involve all layer of gut) | Only surface epithelium intact (Loss of entire crypt with the surface epithelium remaining intact) | 51-75%  (Up to 75% of the tissue effected by disease process) |
| 4 | - | - | Entire crypt and epithelium lost  (Loss of the entire crypt and surface epithelium) | 76-100%  (More Than 75% of the tissue effected by disease process) |

**Supplementary Table 4: List of antibodies used for IHC staining.**

| **Antibody** | **Company** |
| --- | --- |
| Rat Anti Human CD45 Monoclonal Antibody  (Human Specific) | SYSY-HistoSure |
| Rat Anti Human CD3 Monoclonal Antibody  (Human Specific) |  |
| Rat Anti Human FoxP3 Monoclonal Antibody | Invitrogen |
| Rabbit Anti Anti-myeloperoxidase (MPO) Polyclonal Antibody | Abccam |

**Supplementary Table 5: Mean hCD45⁺ engraftment rates and final day Disease Activity Index (DAI) scores across experimental groups**

| Mouse | hCD45 engraftment rate(%) | DAI Final Day |
| --- | --- | --- |
| Normal | 85.62 | 0.00 |
| Vehicle | 94.13 | 12.00 |
| Ozanimod | 56.89 | 5.13 |
| Treg Cell | 73.17 | 2.63 |
| Non-Treg Cell | 87.63 | 7.88 |


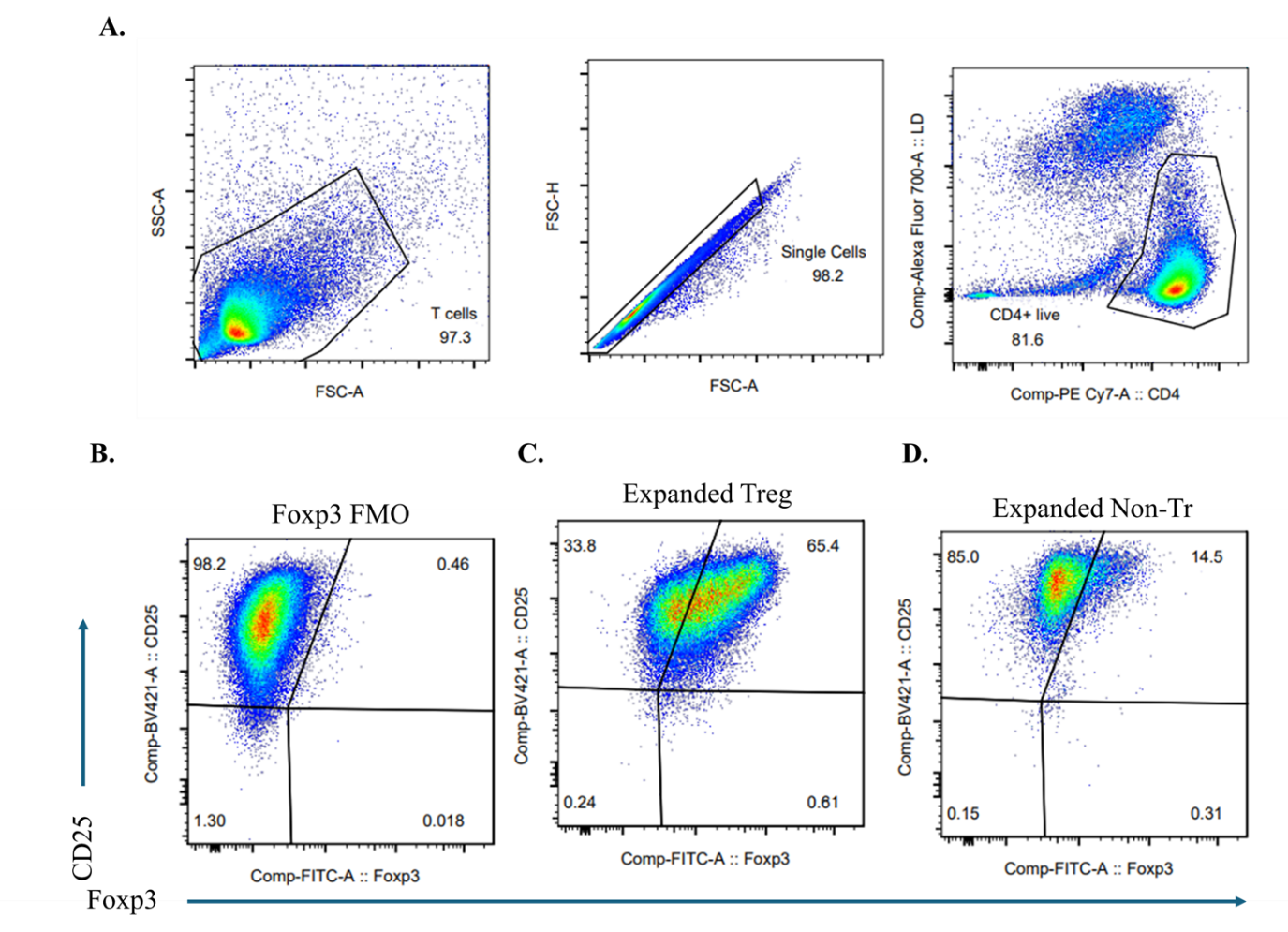


**Supplementary Figure 1. Flow cytometric analysis of expanded Treg and Non-Treg cells.**

Representative flow cytometry gating strategy and analysis of expanded CD4⁺ T cells. (A) Initial gating strategy to identify single cells, T cells, and live CD4⁺ cells. (B) FoxP3 fluorescence minus one (FMO) control, (C) Gating for CD25⁺FoxP3⁺ Treg cells after expansion, and (D) gating for CD25^+^FoxP3⁻ Non-Treg cells. After expansion, 65.4% of live CD4⁺ cells were FoxP3⁺CD25⁺ Treg cells, and 14.5% were Non-Treg cells.


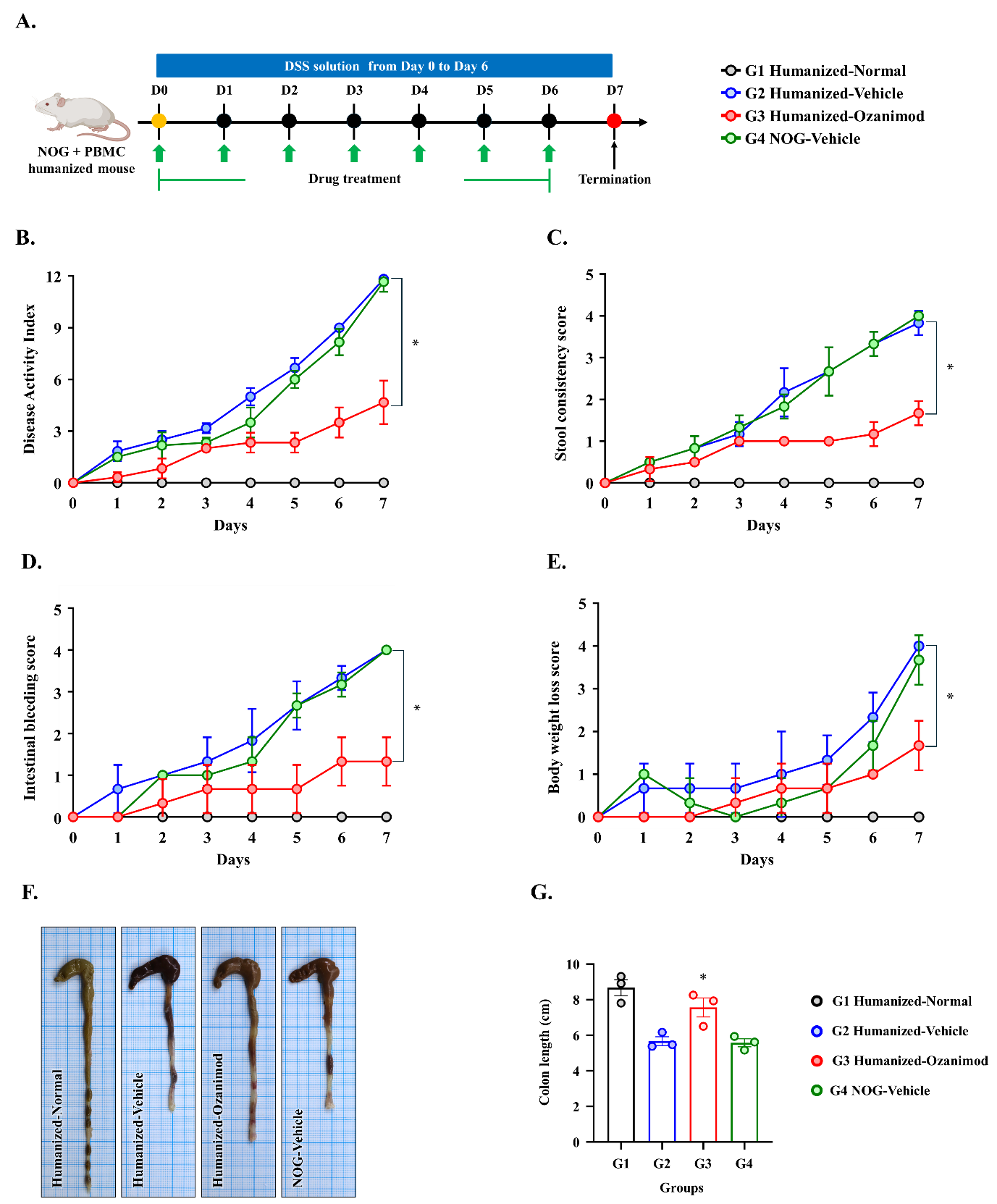


**Supplementary Figure 2. DSS-induced colitis in PBMC-humanized and non-humanized NOG mice.**

(A) Schematic representation of the experimental timeline for DSS-induced colitis. Mice received DSS solution in drinking water from Day 0 to Day 6. Treatments were initiated during DSS exposure as indicated: Humanized-Normal (PBMC-humanized mice without DSS-induced colitis), Humanized-Vehicle (PBMC-humanized mice with DSS-induced colitis, and treated with PBS), Humanized-Ozanimod (PBMC-humanized mice with DSS-induced colitis and treated with 5 mg/kg ozanimod), NOG-Vehicle (non-humanized NOG mice with DSS-induced colitis, and treated with PBS). (B) Disease Activity Index (DAI), (C) stool consistency score, (D) intestinal bleeding score, and (E) body weight loss scores for the different groups. DAI was calculated as the sum of stool consistency, intestinal bleeding, and body weight loss scores. (F) Representative images of colons from each group demonstrating macroscopic changes. (G) Colon length as a measure of colitis severity. Data are presented as mean ± SD (N = 3 per group). *P < 0.05 versus the Humanized-Vehicle group.


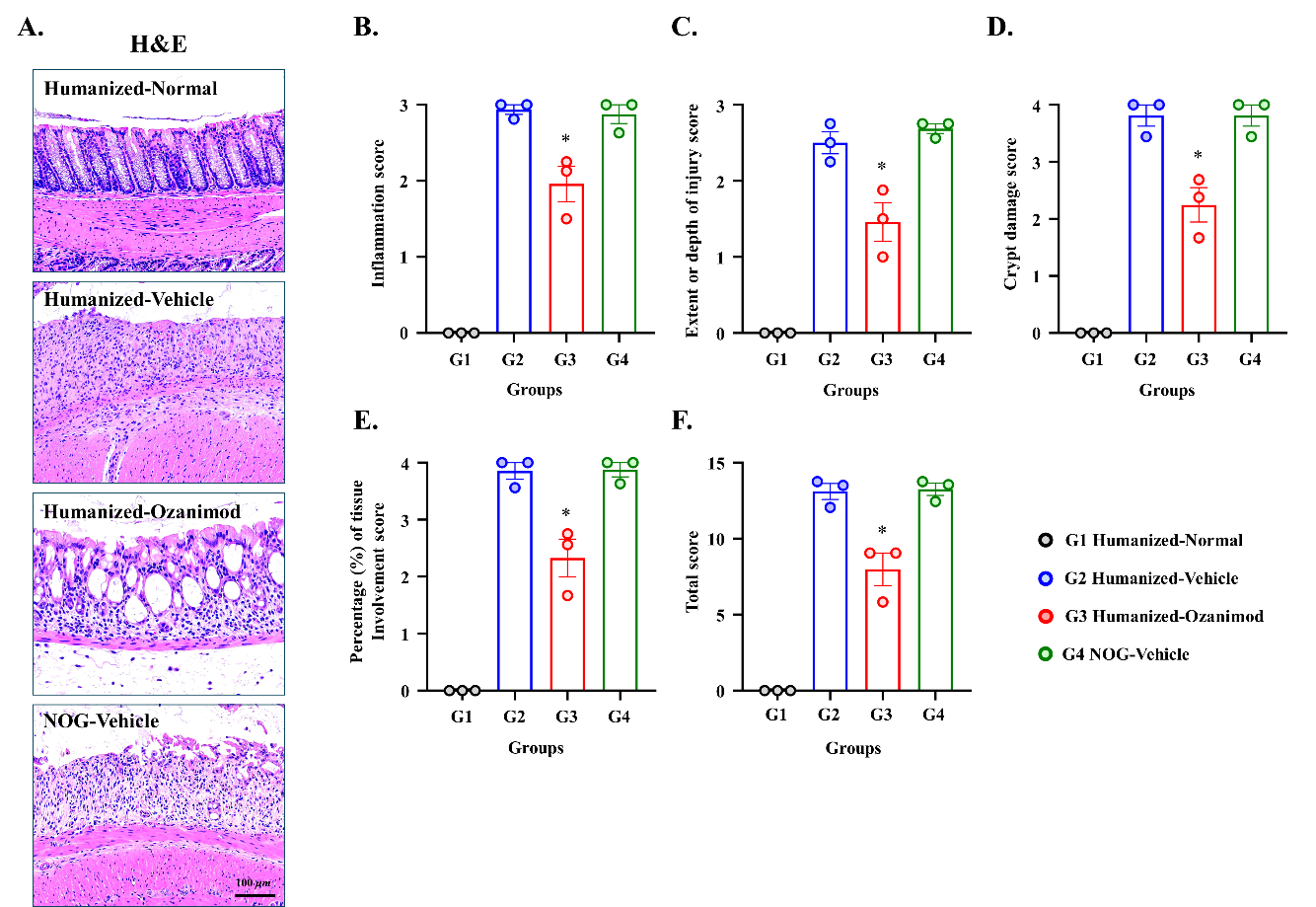


**Supplementary Figure 3. Histopathological evaluation of DSS-induced colitis in humanized and non-humanized NOG mice.**

(A) Representative H&E-stained colon sections from each experimental group collected at Day 7 post-DSS induction. (B–F) Semiquantitative histological scoring was performed by two blinded researchers to assess tissue damage across the following parameters: (B) Inflammation score, (C) Extent or depth of injury score, (D) Crypt damage score, (E) Percentage of tissue involvement, and (F) Cumulative total histological score. The experimental groups included: G1: Humanized-Normal – PBMC-humanized mice without DSS treatment; G2: Humanized-Vehicle – PBMC-humanized mice treated with DSS and injected with PBS; G3: Humanized-Ozanimod – DSS-treated humanized mice receiving 5 mg/kg ozanimod; G4: NOG-Vehicle – Non-humanized NOG mice treated with DSS and PBS. Data are presented as mean ± SD (N = 3 per group). *P < 0.05 versus the Humanized-Vehicle group (G2). Scale bar = 100 µm.
